# Exoscopic Transcortical-Transventricular Approach With Tubular Retractor for Chronic Encapsulated Expanding Hematoma in the Thalamus

**DOI:** 10.7759/cureus.71211

**Published:** 2024-10-10

**Authors:** Katsuya Ueno, Akina Iwasaki, Takuma Maeda, Daisuke Tanikawa, Aoto Shibata, Masahito Kobayashi, Hiroki Kurita

**Affiliations:** 1 Department of Neurosurgery, Saitama Medical University International Medical Center, Hidaka, JPN; 2 Department of Neurosurgery, Saitama Medical University Hospital, Moroyama, JPN

**Keywords:** chronic encapsulated expanding hematoma, exoscope, thalamic lesion, transcortical-transventricular approach, tubular retractor

## Abstract

The feasibility of surgical treatment for hemorrhagic deep thalamic lesions is becoming better understood in line with the improvement in microscopic and endoscopic techniques. However, the indications for and approaches to surgical treatment remain unclear. Herein, we report two cases of chronic encapsulated expanding hematomas (CEEH) in the thalamus resected through an exoscopic transcortical-transventricular approach using a tubular retractor. The first patient presented with progressive right hemiparesis and the second patient exhibited painful left hemidysesthesia. Computed tomography (CT) revealed a high-density mass in the thalamus with perifocal brain edema. Fluid-attenuated inversion recovery magnetic resonance imaging revealed a reticular pattern of mixed hyperintensity and hypointensity, with a thick capsule and mild edematous changes around the hemorrhage in both cases, suggesting CEEH. We performed minimally invasive resection of thalamic CEEHs using an exoscopic transcortical-transventricular approach with the tubular retractor of the ViewSite Brain Access System (Vycor Medical Inc., Boca Raton, FL, US). Corticotomies were made in the left frontal lobe in the first case and the right parietal lobe in the second case. Subtotal resection was achieved without any significant complications. In both cases, the symptoms resolved, and the patients were referred to a rehabilitation hospital with modified Rankin Scale scores of 3 and 2, respectively. An exoscopic transcortical-transventricular approach with a tubular retractor was effective for thalamic lesions with minimal invasiveness and good manipulation of the procedure.

## Introduction

Surgical approaches for thalamic lesions are challenging because of their deep-seated locations surrounded by important structures. However, with advances in neuroimaging and surgical techniques, including microsurgery and endoscopic surgery, surgical resection of thalamic lesions is becoming more feasible [1-3]. In some patients, the prognosis significantly improves with surgical approaches compared with conservative therapy; however, the indications and optimal surgical approaches for surgical treatment remain controversial due to the non-negligible risks of surgical invasion.

Herein, we report two cases of chronic encapsulated expanding hematoma (CEEH) in the thalamus that were surgically treated using an exoscopic transcortical-transventricular approach, which enabled a minimally invasive and well-visualized procedure.

## Case presentation

Case 1

**Figure 1:**
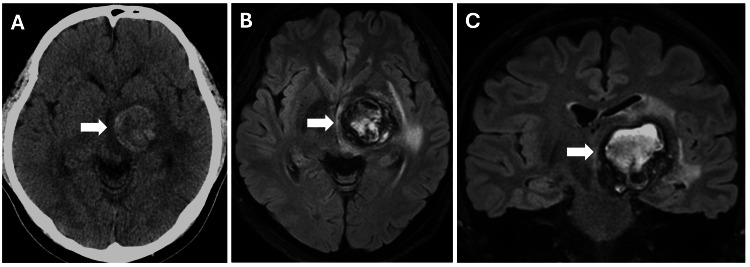
Initial imaging studies (Case 1) Computed tomography on admission demonstrating a left thalamic hemorrhage with a maximum diameter of 3 cm (A, arrow). Fluid-attenuated inversion recovery magnetic resonance imaging showing a reticulated pattern of mixed hyperintensity, hypointensity, and mild edematous changes around the hemorrhage (B and C, arrows).

A 31-year-old man with a history of diabetes mellitus developed a two-month history of clumsiness in the right upper extremity and gait disturbance. On admission, the patient had right hemiparesis with a manual muscle test (MMT) grade of 3. CT revealed a hyperdense mass in the left thalamus with a maximum diameter of 3 cm (Figure 1A). Fluid-attenuated inversion recovery (FLAIR) magnetic resonance imaging (MRI) showed a reticulated pattern of mixed hyper- and hypointensity with a thick capsule and edematous changes around the hemorrhage, suggesting persistent repetitive hemorrhage and worsening edematous changes over time (Figures 1B-1C). Cerebral angiography revealed no evident vascular malformations. The patient was diagnosed with CEEH.

**Figure 2:**
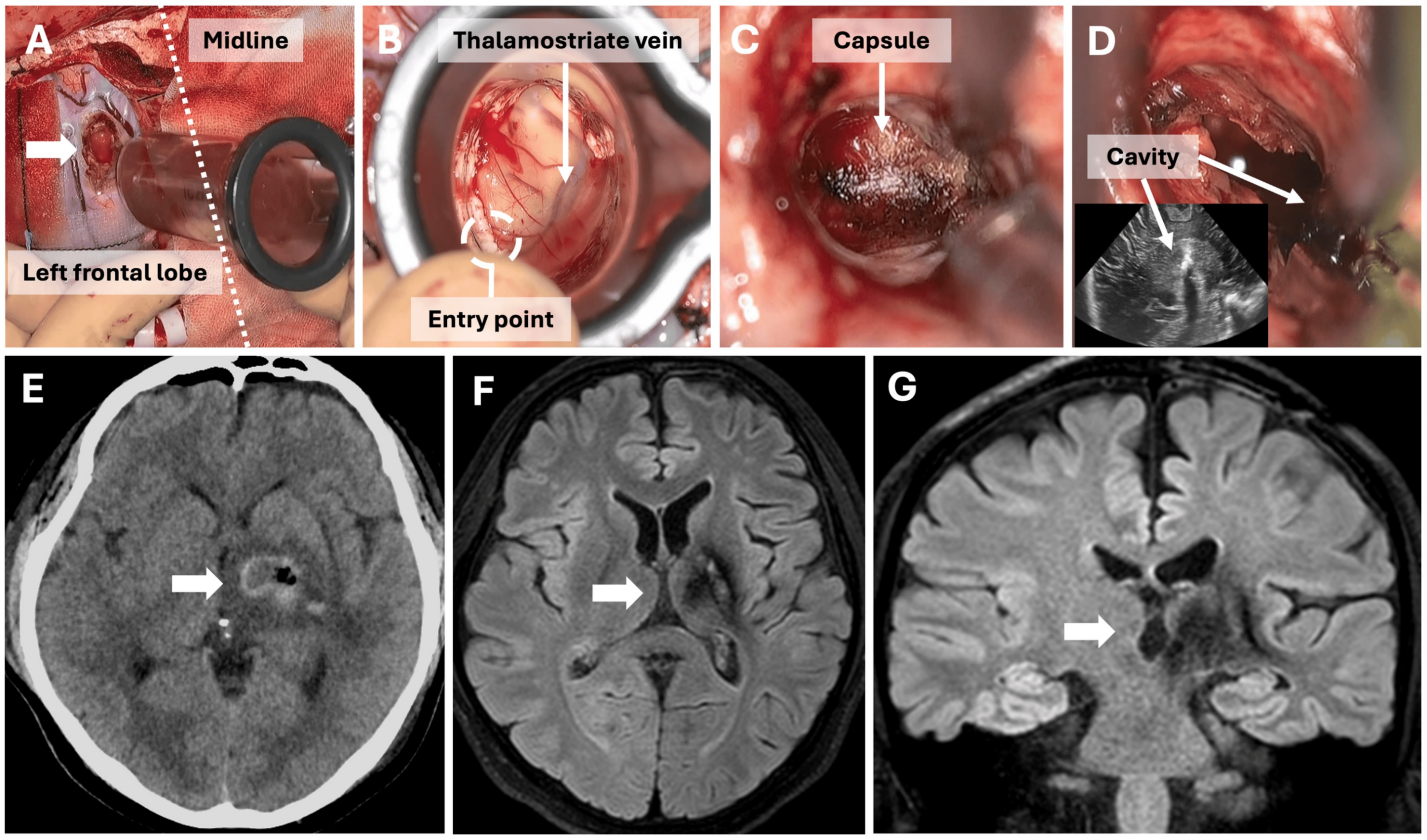
Intraoperative and postoperative images (Case 1) A 1 × 2 cm corticotomy was performed 9 cm parietal and 1.5 cm lateral to the nasion (A, arrow). The ViewSite Brain Access System (21 mm × 7 cm) was inserted into the left lateral ventricle along with the corticotomy, and the entry point into the hemorrhage was determined to be 1 cm lateral to the thalamostriate vein, which was the closest point to the hemorrhage (B). The hematoma capsule and vascular conglomerates were dissected (C arrow). Subtotal removal of the hematoma capsule was confirmed using intraoperative ultrasonography (D arrows). Computed tomography on postoperative day 1 shows a reduction in hematoma with no further bleeding or infarction (E, arrow). Magnetic resonance imaging at the six-month follow-up showing further shrinkage of the residual lesion (F and G, arrows).

The surgical procedure was performed under general anesthesia using motor-evoked potential, somatosensory-evoked potential, and optical navigation. The patient was placed in the supine position and a left frontal craniotomy was performed with a coronal skin incision in the hairline. A corticotomy was performed 9 cm parietal and 1.5 cm lateral to the nasion (Figure 2A). The ViewSite Brain Access System (VBAS; Vycor Medical, Boca Raton, FL, USA; 21 mm × 7 cm) was inserted toward the left lateral ventricle along the corticotomy (Figure 2B). We identified the foramen of Monro, the thalamostriate vein, and the anterior septal vein and determined the entry point into the hemorrhage 1 cm lateral to the thalamostriate vein, which was the closest point to the hemorrhage. The hematoma capsule was fibrotic; hence, mass reduction was performed by sharp dissection and aspiration of the internal hematoma. The capsule and vascular conglomerates were excised and dissected until normal brain tissue was observed (Figure 2C). The hematoma capsule located in the deep lateral part of the thalamus was not dissected because it was in contact with the posterior limb of the internal capsule and midbrain, and only the vascular conglomeration inside the capsule was cauterized. We confirmed subtotal removal of the hematoma capsule using intraoperative ultrasound (Figure 2D). Pathological examination revealed a hematoma capsule with fibrotic changes, granulation, and hemosiderin deposition, leading to the diagnosis of a possible cavernous malformation. Postoperative CT revealed hematoma reduction with no further bleeding or infarction (Figure 2E). One week after surgery, the right hemiparesis improved to MMT grade 4 with rehabilitation. The residual lesion in the deep lateral part of the thalamus was treated with CyberKnife radiotherapy (Accuray Incorporated, Sunnyvale, California, US) with a 14 Gy single fraction to prevent rebleeding. On postoperative day 27, the patient was referred to another hospital for further rehabilitation, with a modified Rankin Scale (mRS) score of 3. CT and FLAIR MRI at the six-month follow-up revealed further shrinkage of the residual lesion (Figures 2F-2G).

Case 2

**Figure 3:**
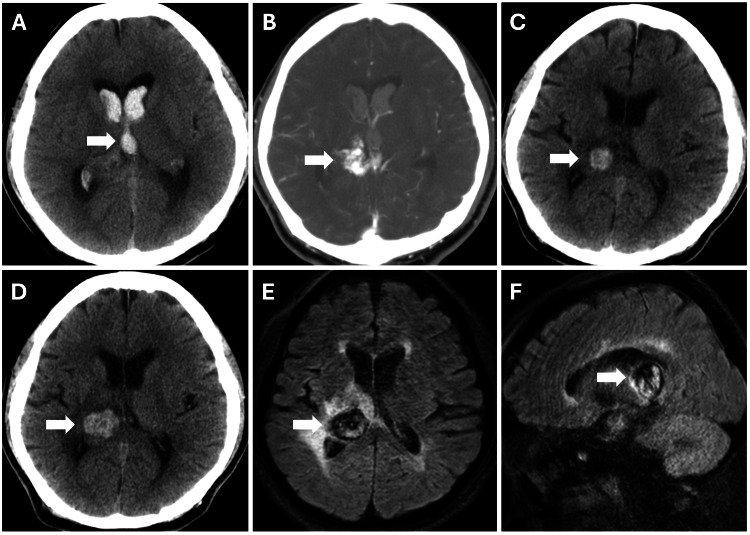
Initial imaging findings (Case 2) Computed tomography (CT) demonstrating an intraventricular hemorrhage (IVH) 14 years previously (A, arrow). CT angiography shows a right thalamic arteriovenous malformation as the cause of IVH (B arrow). CT showing a right thalamic hemorrhage with a maximum diameter of 1.4 cm (C arrow), which increased to 2.4 cm two months later (D arrow). Fluid-attenuated inversion recovery magnetic resonance imaging showing a reticulated pattern of mixed hyperintensity, hypointensity, and mild edematous changes around the hemorrhage (E and F, arrows).

The second patient was a 51-year-old man who had undergone CyberKnife treatment for a right hemorrhagic thalamic arteriovenous malformation (AVM, Spetzler and Martin grade III) 14 years previously (Figures 3A-3B) and had a history of dialysis for renal failure. Four months ago, he developed intractable painful hemidysesthesia in the left hemisphere. CT revealed a right thalamic hemorrhage with a maximum diameter of 1.4 cm (Figure 3C), which increased to 2.4 cm 2 months later (Figure 3D). Fluid-attenuated inversion recovery (FLAIR) MRI showed a reticulated pattern of mixed hyper- and hypointensity with a thick hematoma capsule (7 mm) and perifocal edema, similar to those observed in Case 1 (Figures 3E-3F). Cerebral angiography revealed no residual AVM or other abnormal findings. The patient was diagnosed with CEEH after radiosurgery.

**Figure 4:**
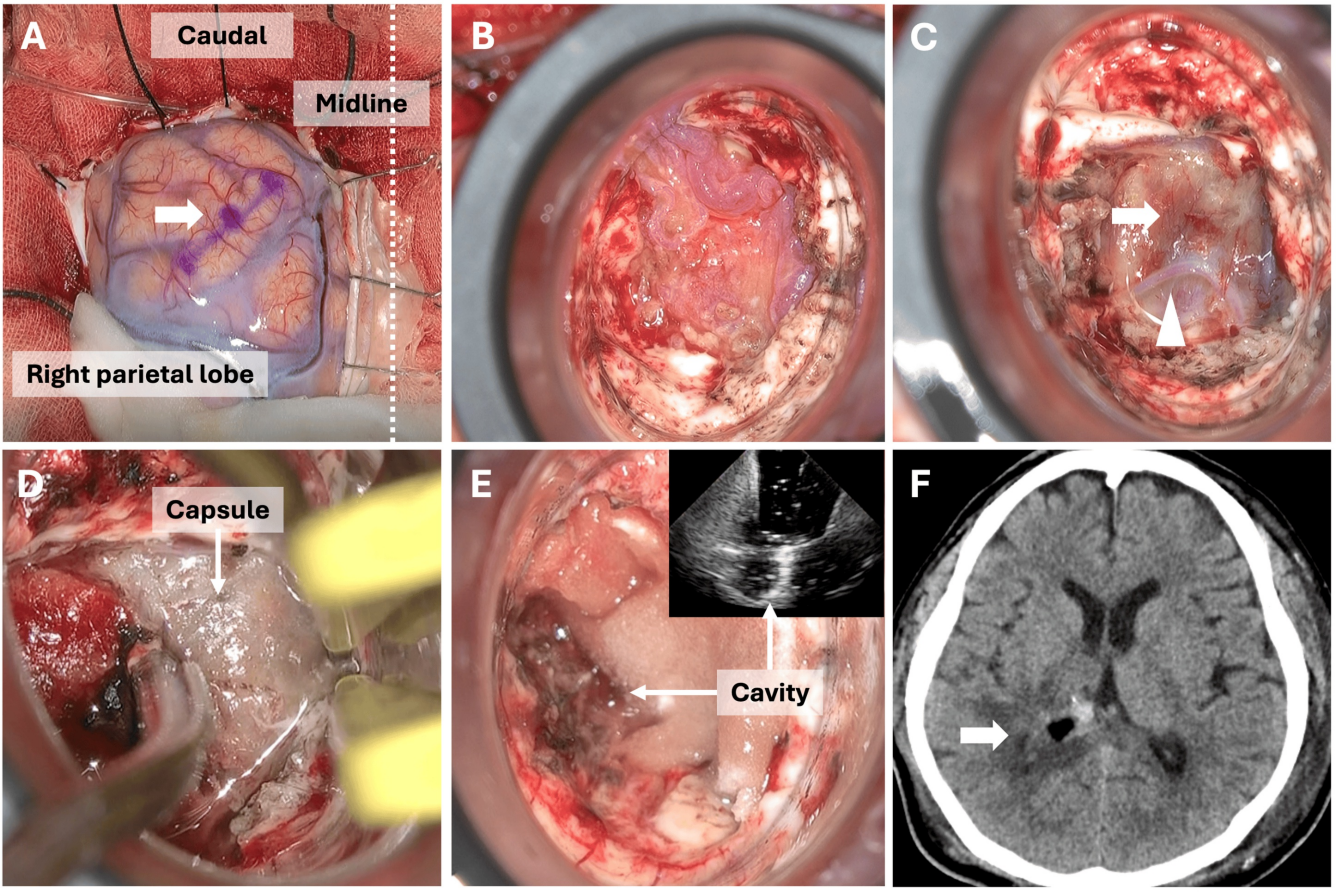
Intraoperative and postoperative images (Case 2) A corticotomy was performed 2 cm lateral to the median in the superior parietal lobule (A, arrow). A ViewSite Brain Access System (21 × 5 cm) was inserted under optical navigation (B). The hematoma capsule (C, arrow) and feeder (C, arrowhead) were identified by removing the ependyma. The hematoma capsule and vascular conglomerates were dissected (D arrow). Subtotal removal of the hematoma capsule was confirmed using intraoperative ultrasonography (E, arrows). Postoperative computed tomography showed complete removal of the hematoma capsule (F, arrow).

Anesthesia and neuromonitoring were consistent with those in Case 1. The patient was placed in a prone position and a U-shaped skin incision was made on the caudal side of the base. An 8 cm x 7.5 cm craniotomy was performed across the superior sagittal sinus (Figure 4A). A corticotomy was performed 2 cm lateral to the midline of the right superior parietal lobe. A VBAS (21 mm × 5 cm) was inserted into the trigone of the right lateral ventricle (Figure 4B). The hematoma capsule was accessed by cauterizing and removing the ependyma at the point closest to the capsule (Figure 4C). The feeder vessels around the hematoma capsule were cauterized, and circumferential dissection of the capsule was performed, removing it piece by piece (Figures 4C-4D). Careful dissection of the deep part of the capsule was necessary because of its proximity to the pyramidal tract. Intraoperative ultrasound confirmed the subtotal removal of the capsule (Figure 4E). Pathological examination revealed an occluded lumen, vitrified vasculature, and capsule-like tissue, leading to the diagnosis of CEEH. Postoperatively, the patient’s dysesthesia resolved and CT showed complete removal of the hematoma capsule (Figure 4F). The patient was transferred to another hospital for further rehabilitation 46 days after the initial surgery with a mRS score of 2.

Written informed consent for publication was obtained from the patients. This research did not require ethics committee approval, as it involved retrospective individual case descriptions.

## Discussion

Herein, surgery for thalamic lesions was successfully performed using an exoscopic transcortical-transventricular approach with VBAS without significant complications. Although conservative treatment is the first-line option for hemorrhagic lesions in deep or complex areas, surgical treatment is becoming more feasible owing to advances in intraoperative neuroimaging and microscopic and endoscopic techniques [1-7]. The principle of the surgical approach for thalamic lesions is to fully expose the target area and minimize the risk of damaging the surrounding vital structures [8].

Because the thalamus is surrounded by important neurological structures, the approach route should be carefully considered based on the location of the hemorrhage. Most of the surface of the thalamus is covered by the internal capsule laterally and by the hypothalamus and mesencephalon inferiorly. However, the superior surface of the thalamus is connected to the floor of the lateral ventricular body, and the medial surface of the thalamus is connected to the lateral walls of the third ventricle, allowing for the transventricular approach [9]. Additionally, the transventricular approach can provide sufficient observation space for resection with minimal damage to the brain parenchyma. Several approaches have been reported for hemorrhagic lesions in the thalamus and basal ganglia based on the extent of the lesions (Table 1) [6,7,9-12]. The transcallosal-transventricular approach is suitable for medial thalamic lesions and reportedly has good surgical results. In cases involving the lateral thalamus or part of the basal ganglia, an anterior contralateral interhemispheric or transinsular approach may be used. However, in contrast to medial thalamic lesions, postoperative neurological findings may deteriorate in some cases of lateral lesions [6]. In particular, the transinsular approach has the risk of damaging the posterior limb of the internal capsule. It is sometimes difficult to reach the lesion in the lateral superior component because of the extent of manipulation and the field of view with the anterior contralateral interhemispheric approach. For lateral posterior lesions, the parieto-occipital transventricular or parietal transsulcal parafascicular approach can be performed; however, there is a risk of damage due to optical radiation [9,11]. The transcortical-transventricular approach is suitable for approaching the lateral posterior component of the thalamus while avoiding damage to critical structures around the thalamus.

**Table 1:** Previous reports on surgical approach for hemorrhagic thalamic lesions ACT; anterior contralateral interhemispheric transcallosal, AIT; anterior ipsilateral interhemispheric transcallosal, CN; cranial nerves, h/h; hemiparesis or hemiparesthesias, N/A; not available, PIT; posterior interhemispheric transcallosal, POT; parietooccipital transventricular, PTi; posterior transinsular, PTPF; parietal trans-sulcal para-fascicular, th; thalamus. *Parenchymal injury or intraoperative hemorrhage

Author & Year	No. of Patients	Location	Approach	Residual Lesion	Complication	Deterioration	Neurological Deficit
Chang et al., 2011 [6]	2	Medial th	AIT	N/A	0	0	None
	1	Medial th	PIT	N/A	0	0	None
	4	Lateral th, basal ganglia	PTi	N/A	2*	1	h/h
Rangel-Castilla et al., 2015 [7]	9	Medial th	AIT	No	0	0	h/h
	17	Lateral th	ACT	No	0	2	h/h, CN Ⅶ
	3	Posterosuperior th	PIT	Yes	0	0	h/h
	4	Lateral posteroinferior th	POT	No	0	1	h/h, CN Ⅲ
Amenta et al., 2016 [9]	1	Posterolateral	PTPF	Yes	0	0	h/h
Ding et al., 2017 [10]	1	Anterior medial th	ACT	No	0	0	Ataxia
Amoo et al., 2021 [11]	1	Posterolateral	PTPF	Yes	0	0	0
Catapano et al., 2023 [12]	6	Anterior th	AIT	N/A	N/A	0	N/A
	18	Medial th	ACT	N/A	N/A	2	N/A
	10	Lateral th	PTi	N/A	N/A	0	N/A

Herein, VBAS was used to obtain sufficient surgical space and minimize parenchymal damage. Because tubular retractors radially allocate pressure and widen rather than cut white matter tracts, they are considered to cause less damage to normal brain tissue than conventional blade-type retractors [13]. They also allow flexible rotation and angular adjustment without exerting extra pressure on brain tissue and provide a wider field of view than microscopes alone or endoscopes [14]. In our cases, the VBAS was inserted in a straight line in the cortex up to the anterior horn and trigon of the ventricle to reduce the burden on the surrounding brain while maintaining the manipulation space. In addition, we used a transventricular approach with the shortest possible distance from the ventricles to the hematoma cavity, avoiding damage to the normal brain tissue and vital structures, including the pyramidal tracts. Microscope-assisted surgery with tubular retractors resulted in better surgical outcomes than endoscope-assisted surgery owing to a wider 3D field of view [15]. The use of an exoscope may enhance this advantage by allowing the light source to penetrate deeper without forcing the surgeon into an uncomfortable posture. The exoscope also provides a free visual axis with a small body and high image quality [16].

A chronic encapsulated expanding hematoma is a rare hemorrhagic lesion characterized by the presence of a fibrotic capsule. It is sometimes associated with AVM, cavernous malformations, capillary telangiectasia, microaneurysm, and stereotactic radiosurgery [17]. A fibrotic capsule due to repeated hemorrhage makes surgery more challenging [1]. Repeated bleeding also destroys surgical planes, making it difficult to separate reactive tissue components from lesions [2]. It is controversial whether capsule removal is necessary, especially in deep-seated lesions, although it has been suggested to prevent recurrence [17]. Further prospective studies are warranted to determine the optimal timing and therapeutic approach for deep-seated CEEH.

## Conclusions

We demonstrated the feasibility of an exoscopic transcortical-transventricular approach using VBAS for thalamic CEEH. This approach provides a sufficient observation space with minimal damage to the brain parenchyma. In symptomatic lesions, surgical intervention for CEEH using a modified approach may be a potential therapeutic option, even for deep-seated lesions.
